# ZEB1 Expression in Endometrial Biopsy Predicts Lymph Node Metastases in Patient with Endometrial Cancer

**DOI:** 10.1155/2014/680361

**Published:** 2014-12-03

**Authors:** Gang Feng, Xiangming Wang, Xiaozhi Cao, Lijuan Shen, Jiansheng Zhu

**Affiliations:** ^1^Clinical Genetics Laboratory, The First Affiliated Hospital of Wannan Medical College, Wuhu, Anhui 241001, China; ^2^Department of Pathology, The First Affiliated Hospital of Wannan Medical College, Wuhu, Anhui 241001, China; ^3^Department of Pathology, The Second Affiliated Hospital of Wannan Medical College, Wuhu, Anhui 241001, China; ^4^Department of Pathology, Wuhu Hospital of Traditional Chinese Medicine, Wuhu, Anhui 241001, China; ^5^Molecular Pathological Laboratory, Maternal and Child Health Hospital of Anhui Province, Anhui Medical University, Hefei, Anhui 230001, China

## Abstract

*Purpose*. The purpose of this study was to analyze the expression of zinc-finger E-box-binding homeobox 1 (ZEB1) in endometrial biopsy and its correlation with preoperative characteristics, including lymph node metastases in patient with endometrial cancer. *Methods*. Using quantitative RT-PCR, ZEB1 expressions in endometrial biopsy from 452 patients were measured. The relationship between ZEB1 expression and preoperative characteristics was analyzed. *Results*. ZEB1 expressions were significantly associated with subtype, grade, myometrial invasion, and lymph node metastases. Lymph node metastases could be identified with a sensitivity of 57.8% at specificity of 74.1% by ZEB1 expression in endometrial biopsy. Based on combination of preoperative characteristics and ZEB1 expression, lymph node metastases could be identified with a sensitivity of 62.1% at specificity of 96.2% prior to hysterectomy. *Conclusion*. ZEB1 expression in endometrial biopsy could help physicians to better predict the lymph node metastasis in patients with endometrial cancer prior to hysterectomy.

## 1. Introduction

Endometrial cancer is the most common malignancy of the female reproductive tract and the fourth most common cancer overall [1]. The incidence of endometrial cancer is rising due to the global obesity epidemic, increased life expectancy, and the falling rate of hysterectomy for benign disease [2]. Disease progression is usually slow, especially for endometrioid adenocarcinoma. Overall, the disease has a good prognosis [3]. Endometrial cancer is usually successfully treated with surgery and/or radiotherapy. However, for patients with advanced or recurrent endometrial cancer, fewer treatment options are available [4].

Lymph node metastasis is usually identified by pathological report from systematic lymphadenectomy [5]. However, systematic lymphadenectomy has recently been questioned, particularly as pelvic lymphadenectomy has no impact on overall survival of patients with early stage endometrial cancer, whereas it increases postoperative complication rates [6, 7]. Therefore, French guidelines have recently been modified and pelvic lymphadenectomy is no longer recommended in women with low- or intermediate-risk endometrial cancer [8]. However, a multicentre study recently demonstrated that lymph node metastases are also found in 10% of women with low-risk and 15% of women with intermediate-risk endometrial cancer by sentinel lymph node biopsy [9].

Preoperative clinicopathological characteristics and depth of myometrial invasion are usually for decisions of different risk endometrial cancer. However, accuracy of formula which is based on the preoperative characteristics has not been externally validated. Therefore, a biomarker for better risk stratification in endometrial cancer is needed to improve individualized primary treatment and help avoid over- or undertreatment of patient with endometrial cancer.

Zinc-finger E-box-binding homeobox 1 (ZEB1, also known as dEF1, Nil-2-a, Tcf8, Bzp, Areb6, Meb1, Zfhx1a, and Zfhep) has been identified as a transcriptional factor which could induce epithelial to mesenchymal transition (EMT) [10, 11]. Spaderna et al. described that EMT-inducing transcriptional repressor ZEB1 promotes colorectal cancer cell metastasis [12]. Bae et al. demonstrated that the elevated invasiveness was a result of the activated EGFR-MEK/ERK signaling, which in turn led to ZEB1 induction in non-small-cell lung cancer [13]. Hashiguchi et al. demonstrated that positive ZEB1 expression was correlated with poor prognosis in hepatocellular carcinoma patients [14]. The implication of ZEB1 in melanoma biological processes, such as invasion and metastasis, has also been described [15]. In particular, ZEB1 was not expressed in the normal endometrial epithelium and was aberrantly expressed in tumor epithelial cells of aggressive endometrial cancers [16].

The purpose of this study was to analyze the expression of ZEB1 in endometrial biopsy and its correlation with preoperative characteristics, including lymph node metastases in patient with endometrial cancer. We also evaluated whether ZEB1 expression in endometrial biopsy could predict lymph node metastases in patients with endometrial cancer.

## 2. Materials and Methods

### 2.1. Patients

This cohort study included patients with endometrial cancer between June 2012 and June 2014 from four centres. All patients with endometrial cancer were diagnosed by endometrial biopsy with Pipelle (Endocurrette, Midvale, Utah, USA). Prior to hysterectomy, the depth of myometrial invasion was evaluated by T2 weighted imaging combined with diffusion weighted imaging (GE Signa EXCITE Twin Speed HD, 1.5 T system). Each patient had no hormonal, cytotoxic, or radiation therapy before hysterectomy. All patients underwent total abdominal hysterectomy, with bilateral salpingo-oophorectomy and pelvic lymphadenectomy. During the surgery, para-aortic lymphadenectomy was done if suspicious nodes were detected by frozen section examination.

The preoperative histological subtype and grade were abstracted from the pathological analysis of endometrial samples. Patients were identified preoperatively as low-risk (endometrioid, grade 1 or 2, myometrial invasion <50%) or high-risk (no endometrioid, grade 3, myometrial invasion >50%) for lymph node metastases. If the patient did not meet the above criteria, she was viewed as intermediate-risk for lymph node metastases.

The clinical stage was assessed based on the evaluation of the hysterectomy specimens according to the International Federation of Gynecology and Obstetrics (FIGO) 2009 system. Patients were also divided into three risk factor groups: low-risk (endometrioid FIGO stage IA grade 1 or 2); intermediate-risk (endometrioid FIGO stage IA grade 3 or FIGO stage IB grade 1 or 2); high-risk (nonendometrioid, endometrioid FIGO stage IB G3, or FIGO II and higher) [17].

The research protocol was approved by Wannan Medical College and Anhui Medical University.

### 2.2. ZEB1 mRNA Detection

Cytologic sampling was also carried out using Pipelle. Total RNA was extracted using RNeasy Mini Kit (Qiagen S. A.) according to the manufacturer's protocol. Reverse transcription was performed with the Super-Script First-Strand Synthesis System for reverse transcriptase-polymerase chain reaction (RT-PCR) (Invitrogen), using 1.0 *μ*g of total RNA and following manufacturer's instructions.

The primers for the ZEB1 are forward: 5′-TCC ATG CTT AAG AGC GCT AGC T-3′ and reverse: 5′-ACC GTA GTT GAG TAG GTG TAT GCC A-3′. The primers for the glyceraldehyde-3-phosphate dehydrogenase (GAPDH) are forward: 5′-ACG GAT TTG GTC GTA TTG GGC G-3′ and reverse: 5′-CTC CTG GAA GAT GGT GAT GG-3′. Quantitative real-time PCR was performed on a Roche LightCycler 480-II. Each 20 *μ*L reaction mixture contains 10 *μ*L of 2X Roche SYBR Green I Master Mixes, 2 *μ*L of complementary deoxyribonucleic acid template, and 100 nM of each primer. The thermal profile was a first denaturation step at 95°C for 10 minutes, followed by 35 cycles at 95°C for 10 seconds, 55°C for 15 seconds, and 72°C for 15 seconds. Melting curve analysis was performed to confirm PCR product's specificity. A standard curve was generated using the fluorescence data from the 10-fold serial dilutions of known quantities of control plasmid for human ZEB1 and human GAPDH. The amount of ZEB1 was normalized to the amount of GAPDH. All samples were analyzed in triplicate.

### 2.3. Statistics

ZEB1 expressions were expressed as Mean ± SD. Comparisons between preoperative and postoperative characteristics were made using Chi-square test. Comparisons between preoperative characteristics and ZEB1 expressions were made using Mann-Whitney* U* test. Receiver operator curves (ROC) were used to compare the ability to identify patients with lymph node metastasis by ZEB1 expression. Differences were considered significant at a level of *P* < 0.05. All statistical analyses were performed using the SPSS 13.0 statistical package.

## 3. Results

A total of 452 patients were included in the study. The preoperative and postoperative characteristics were reported in Table 1. There were no statistically significant differences between the preoperative and postoperative characteristics (grade, subtype, and myometrial invasion) (Table 1).

The median of ZEB1 expression in endometrial biopsy was 3.76 (range 1.54–8.37). The association between ZEB1 expression and preoperative characteristics was shown in Table 2. Age did not influence the ZEB1 expression (*P* = 0.064). The ZEB1 expression in endometrial biopsy significantly associated with subtype (*P* = 0.031), grade (*P* = 0.022), and myometrial invasion (*P* = 0.014).

Among all patients, 58 patients with lymph node metastasis had been confirmed by final pathological reports. The numbers of lymph node metastases according to low-, intermediate-, and high-risk groups were shown in Table 3. According to final pathological reports, 20 patients with lymph node metastasis had been found in low- or intermediate-risk group (*n* = 409) and 38 patients with lymph node metastasis had been found in high-risk group (*n* = 43). Lymph node metastases could be identified with a sensitivity of 65.5% at specificity of 98.7% by postoperative characteristics. In preoperative low- or intermediate-risk group (*n* = 395), lymph node metastasis had been found in 26 patients. 32 patients with lymph node metastasis were found in preoperative high-risk group (*n* = 57). Lymph node metastases could be identified with a sensitivity of 55.2% at specificity of 93.7% by preoperative characteristics (Table 4).

**Figure 1:**
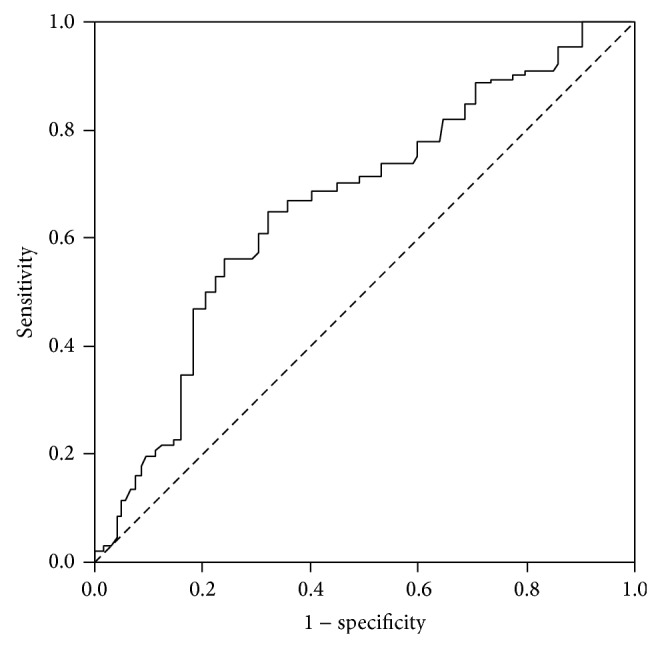
Receiver operating characteristic (ROC) curve of ZEB1 expression in endometrial biopsy for predicting lymph node metastasis in patient with endometrial cancer. The area under the curve (AUC) was 0.657 (95% CI: 0.541–0.774). The best cut-off value was 4.92 (sensitivity: 57.8%; specificity: 74.1%).

Between ZEB1 expression in patients with lymph node metastasis (5.31 ± 3.15) and without lymph node metastasis (3.37 ± 3.02), a significant difference has been found (*P* < 0.001). ROC analyses of ZEB1 expression in patients with and without lymph node metastasis are shown in Figure 1. In this study population, the best cut-off point for ZEB1 expression was 4.92. ZEB1 expression greater than 4.92 demonstrated a sensitivity and specificity of 57.8% and 74.1%, respectively, for lymph node metastasis (ROC AUC = 0.657; 95% CI, 0.541–0.774). Based on the combination of preoperative characteristics and ZEB1 expression, lymph node metastases could be identified with a sensitivity of 62.1% at specificity of 96.2% (Table 4).

## 4. Discussion

In 1988, the International Federation of Obstetrics and Gynecology recommended surgical staging for endometrial cancer patients. However, 25 years later, the role of lymph node dissection remains controversial. The findings of two large independent randomized trials showed that there was no interest from a therapeutic point of view of achieving the pelvic lymphadenectomy when the cancer is confined to the uterus [6, 7]. Theoretically, lymphadenectomy may help identify patients with metastatic dissemination, who may benefit from adjuvant therapy, thus reducing radiation related morbidity. Furthermore, lymphadenectomy may eradicate metastatic disease [18]. Some studies had also shown a possible isolated invasion of para-aortic lymph nodes without pelvic involvement in cases of high-risk tumors [19, 20].

In present study, we estimated the risk for lymph node metastases in 452 patients with endometrial cancer. Based on final pathological reports, 90.5% of endometrial cancer patients were at low- or intermediate-risk for lymph node metastases and the rate of lymph node metastases was only 4.9%. Lymph node metastases could be identified with a sensitivity of 65.5% at specificity of 98.7% by postoperative characteristics in our study. However, this strategy was based on definitive pathological results and was only applicable once surgery had been performed.

Reliable identification of endometrial cancer patients at low-risk for lymph node metastases before surgery remains challenging, and thus the decision to perform a lymphadenectomy is often based on intraoperative frozen section (IFS) to assess histological grade and depth of myometrial invasion. Several investigators had suggested that IFS was an accurate and useful tool to guide intraoperative decision making for surgical staging in endometrial cancer [21, 22]. In contrast, several others had presented data that question the reliability of IFS [23–25]. This ambivalence was reflected in a survey of gynecologic oncologists, in which half of all responders reported that they rarely use IFS to guide their decision to perform lymphadenectomy in endometrial cancer [26].

Endometrial biopsy is the cornerstone of diagnostics of endometrial cancer and the first step of treatment algorithm planning for primary surgical treatment [27]. Although some discrepancies between the preoperative and postoperative characteristics were observed concerning the grade, subtype, and myometrial invasion, we had not found statistically significant differences in these three parameters in our study.

Based on preoperative pathological reports, 87.4% of endometrial cancer patients were at low- or intermediate-risk for lymph node metastases and the rate of lymph node metastases was 6.6%. Lymph node metastases could be identified with a sensitivity of 55.2% at specificity of 93.7% by preoperative characteristics from the pathological analysis of endometrial samples. Although sensitivity and specificity for prediction of lymph node metastases by preoperative characteristics were worse than these by postoperative characteristics, our finding also suggested that the preoperative characteristics could be used to estimate the risk of lymph node metastasis in patients with endometrial cancer.

Higher expression of ZEB1 associated with higher aggressive capacity, poor differentiation, development of metastases, and poor clinical prognosis had recently been revealed in endometrial cancers, colorectal carcinomas, and prostate cancer [15, 28, 29]. In highly migratory and aggressive cell line of endometrial cancer, reduction of ZEB1 expression could result in reduced migratory capacity of cell [30].

As preoperative endometrial biopsy-based assay would be the least invasive and most cost-effective approach, we measured ZEB1 expressions in endometrial biopsies. We observed that histological subtype, grade, and myometrial invasion significantly associated with ZEB1 expression. We also found that ZEB1 expressions in patients with lymph node metastasis were significantly higher than those in patients without lymph node metastasis. Lymph node metastases could be identified with a sensitivity of 57.8% at specificity of 74.1% by ZEB1 expression in endometrial biopsy. Furthermore, lymph node metastases could be identified with a sensitivity of 62.1% at specificity of 96.2% by combination of preoperative characteristics and ZEB1 expression. Our finding suggested that ZEB1 expression could provide additional information which enhances risk assessment greatly prior to hysterectomy.

Our study has several possible limitations. Although the incidence of isolated para-aortic nodal metastasis in patients with negative pelvic nodal metastasis was approximately 1% [31], the fact that only part of patients underwent both pelvic and para-aortic node removal during surgery might influence our results. Although the recruitment protocol and pathological classification is same, the absence of central pathology review and discrepancies between different pathologists also might influence our results. Additionally, other factors that can identify those low-risk patients, such as endometrioid cell type, well or moderate differentiations, and inner half invasion, were not studied in this study. However, we feel that our results are promising enough to encourage further research. The large-scale prospective validation studies are required in order to confirm our present findings.

## 5. Conclusion

ZEB1 expression in endometrial biopsy could help physicians to better predict the lymph node metastasis in patients with endometrial cancer prior to hysterectomy.

## Figures and Tables

**Table 1:** The association between preoperative and postoperative characteristics.

Characteristics	Preoperative	Postoperative	*P* value
Age at diagnosis (years)			
Mean ± SD	63.9 ± 8.7		
Range	47–73		
FIGO stage			
I-II		418	
III-IV		34	
Histological subtype			0.224
Endometrioid	401	412	
Nonendometrioid	51	40	
Histological grade			0.213
Grade 1	219	231	
Grade 2	171	176	
Grade 3	62	45	
Myometrial invasion			0.288
No or <50%	381	369	
≥50%	71	83	

**Table 2:** The association between ZEB1 expression in endometrial biopsy and preoperative characteristics.

	Number	ZEB1 expression (median ± SD)	*P* value
Age			0.064
<60	87	3.25 ± 2.78	
≥60	365	3.88 ± 3.14	
Histologic subtype			0.031
Endometrioid	401	3.65 ± 2.41	
Nonendometrioid	51	4.88 ± 3.53	
Histopathologic grade			0.022
Grades 1 + 2	390	3.66 ± 2.94	
Grade 3	62	5.06 ± 3.37	
Myometrial invasion			0.014
No or <50%	381	3.59 ± 2.98	
≥50%	71	4.66 ± 3.43	

**Table 3:** The numbers of lymph node metastases in different risk groups.

	Preoperative	Lymph node metastasis	Postoperative	Lymph node metastasis
High-risk group	57	32	43	38

Intermediate-risk group	157	19	112	12

Low-risk group	238	7	297	8

**Table 4:** Sensitivity, specificity, PPV, and NPV for predicting lymph node metastasis.

	Postoperative characteristics	Preoperative characteristics	Preoperative characteristics and ZEB1 expression
True positive	38	32	36
True negative	389	369	379
False positive	5	25	15
False negative	20	26	22
Sensitivity	65.5%	55.2%	62.1%
Specificity	98.7%	93.7%	96.2%
PPV	88.4%	56.1%	70.6%
NPV	95.1%	93.4%	94.5%

## References

[1] Siegel R., Naishadham D., Jemal A. (2012). Cancer statistics, 2012. *CA Cancer Journal for Clinicians*.

[2] Yu F., Jiang Q., Zhou Y., Yang Z., Yu X., Wang H., Liu Z., Wang L., Fang W., Guo S. (2012). Abnormal expression of matrix metalloproteinase-9 (MMP9) correlates with clinical course in Chinese patients with endometrial cancer. *Disease Markers*.

[3] Odagiri T., Watari H., Hosaka M., Mitamura T., Konno Y., Kato T., Kobayashi N., Sudo S., Takeda M., Kaneuchi M., Sakuragi N. (2011). Multivariate survival analysis of the patients with recurrent endometrial cancer. *Journal of Gynecologic Oncology*.

[4] Bakkum-Gamez J. N., Gonzalez-Bosquet J., Laack N. N., Mariani A., Dowdy S. C. (2008). Current issues in the management of endometrial cancer. *Mayo Clinic Proceedings*.

[5] Creasman W. (2009). Revised FIGO staging for carcinoma of the endometrium. *International Journal of Gynecology and Obstetrics*.

[6] Panici P. B., Basile S., Maneschi F., Lissoni A. A., Signorelli M., Scambia G., Angioli R., Tateo S., Mangili G., Katsaros D., Garozzo G., Campagnutta E., Donadello N., Greggi S., Melpignano M., Raspagliesi F., Ragni N., Cormio G., Grassi R., Franchi M., Giannarelli D., Fossati R., Torri V., Amoroso M., Crocè C., Mangioni C. (2008). Systematic pelvic lymphadenectomy vs no lymphadenectomy in early-stage endometrial carcinoma: randomized clinical trial. *Journal of the National Cancer Institute*.

[7] Kitchener H., Swart A. M., Qian Q., Amos C., Parmar M. K. (2009). Efficacy of systematic pelvic lymphadenectomy in endometrial cancer (MRC ASTEC trial): a randomised study. *The Lancet*.

[8] Querleu D., Planchamp F., Narducci F., Morice P., Joly F., Genestie C., Haie-Meder C., Thomas L., Quénel-Tueux N., Daraï E., Dorangeon P.-H., Marret H., Taïeb S., Mazeau-Woynar V. (2011). Clinical practice guidelines for the management of patients with endometrial cancer in france recommendations of the institut national du cancer and the société française d'oncologie gynécologique. *International Journal of Gynecological Cancer*.

[9] Ballester M., Dubernard G., Lécuru F., Heitz D., Mathevet P., Marret H., Querleu D., Golfier F., Leblanc E., Rouzier R., Daraï E. (2011). Detection rate and diagnostic accuracy of sentinel-node biopsy in early stage endometrial cancer: a prospective multicentre study (SENTI-ENDO). *The Lancet Oncology*.

[10] Vandewalle C., Van Roy F., Berx G. (2009). The role of the ZEB family of transcription factors in development and disease. *Cellular and Molecular Life Sciences*.

[11] Sánchez-Tilló E., Lázaro A., Torrent R., Cuatrecasas M., Vaquero E. C., Castells A., Engel P., Postigo A. (2010). ZEB1 represses E-cadherin and induces an EMT by recruiting the SWI/SNF chromatin-remodeling protein BRG1. *Oncogene*.

[12] Spaderna S., Schmalhofer O., Wahlbuhl M., Dimmler A., Bauer K., Sultan A., Hlubek F., Jung A., Strand D., Eger A., Kirchner T., Behrens J., Brabletz T. (2008). The transcriptional repressor ZEB1 promotes metastasis and loss of cell polarity in cancer. *Cancer Research*.

[13] Bae G.-Y., Choi S.-J., Lee J.-S., Jo J., Lee J., Kim J., Cha H.-J. (2013). Loss of E-cadherin activates EGFR-MEK/ERK signaling, which promotes invasion via the ZEB1/MMP2 axis in non-small cell lung cancer. *Oncotarget*.

[14] Hashiguchi M., Ueno S., Sakoda M., Iino S., Hiwatashi K., Minami K., Ando K., Mataki Y., Maemura K., Shinchi H., Ishigami S., Natsugoe S. (2013). Clinical implication of ZEB-1 and E-cadherin expression in hepatocellular carcinoma (HCC). *BMC Cancer*.

[15] Dou J., He X., Liu Y. (2014). Effect of downregulation of vimentin expression, tumour migration and tumourigenicity of melanoma B16F10 cells and CSCs. *Cell Biology International*.

[16] Spoelstra N. S., Manning N. G., Higashi Y., Darling D., Singh M., Shroyer K. R., Broaddus R. R., Horwitz K. B., Richer J. K. (2006). The transcription factor ZEB1 is aberrantly expressed in aggressive uterine cancers. *Cancer Research*.

[17] Colombo N., Preti E., Landoni F., Carinelli S., Colombo A., Marini C., Sessa C. (2011). Endometrial cancer: ESMO clinical practice guidelines for diagnosis, treatment and follow-up. *Annals of Oncology*.

[18] Bogani G., Dowdy S. C., Cliby W. A., Ghezzi F., Rossetti D., Mariani A. (2014). Role of pelvic and para-aortic lymphadenectomy in endometrial cancer: current evidence. *Journal of Obstetrics and Gynaecology Research*.

[19] Mariani A., Dowdy S. C., Cliby W. A., Gostout B. S., Jones M. B., Wilson T. O., Podratz K. C. (2008). Prospective assessment of lymphatic dissemination in endometrial cancer: a paradigm shift in surgical staging. *Gynecologic Oncology*.

[20] Todo Y., Kato H., Kaneuchi M., Watari H., Takeda M., Sakuragi N. (2010). Survival effect of para-aortic lymphadenectomy in endometrial cancer (SEPAL study): a retrospective cohort analysis. *The Lancet*.

[21] Shim J. U., Rose P. G., Reale F. R., Soto H., Tak W. K., Hunter R. E. (1992). Accuracy of frozen-section diagnosis at surgery in clinical stage I and II endometrial carcinoma. *The American Journal of Obstetrics and Gynecology*.

[22] Quinlivan J. A., Petersen R. W., Nicklin J. L. (2001). Accuracy of frozen section for the operative management of endometrial cancer. *British Journal of Obstetrics and Gynaecology*.

[23] Case A. S., Rocconi R. P., Straughn J. M., Conner M., Novak L., Wang W., Huh W. K. (2006). A prospective blinded evaluation of the accuracy of frozen section for the surgical management of endometrial cancer. *Obstetrics and Gynecology*.

[24] Frumovitz M., Slomovitz B. M., Singh D. K., Broaddus R. R., Abrams J., Sun C. C., Bevers M., Bodurka D. C. (2004). Frozen section analyses as predictors of lymphatic spread in patients with early-stage uterine cancer. *Journal of the American College of Surgeons*.

[25] Kumar S., Bandyopadhyay S., Semaan A., Shah J. P., Mahdi H., Morris R., Munkarah A., Ali-Fehmi R. (2011). The role of frozen section in surgical staging of low risk endometrial cancer. *PLoS ONE*.

[26] Soliman P. T., Frumovitz M., Spannuth W., Greer M. J., Sharma S., Schmeler K. M., Ramirez P. T., Levenback C. F., Ramondetta L. M. (2010). Lymphadenectomy during endometrial cancer staging: practice patterns among gynecologic oncologists. *Gynecologic Oncology*.

[27] Dinkelspiel H. E., Wright J. D., Lewin S. N. (2013). Contemporary clinical management of cancer. *Obstetrics and Gynecology International*.

[28] Spaderna S., Schmalhofer O., Hlubek F., Berx G., Eger A., Merkel S., Jung A., Kirchner T., Brabletz T. (2006). A transient, EMT-linked loss of basement membranes indicates metastasis and poor survival in colorectal cancer. *Gastroenterology*.

[29] Graham T. R., Zhau H. E., Odero-Marah V. A., Osunkoya A. O., Kimbro K. S., Tighiouart M., Liu T., Simons J. W., O'Regan R. M. (2008). Insulin-like growth factor-I—dependent up-regulation of ZEB1 drives epithelial-to-mesenchymal transition in human prostate cancer cells. *Cancer Research*.

[30] Singh M., Spoelstra N. S., Jean A., Howe E., Torkko K. C., Clark H. R., Darling D. S., Shroyer K. R., Horwitz K. B., Broaddus R. R., Richer J. K. (2008). ZEB1 expression in type I vs type II endometrial cancers: a marker of aggressive disease. *Modern Pathology*.

[31] Abu-Rustum N. R., Gomez J. D., Alektiar K. M., Soslow R. A., Hensley M. L., Leitao M. M., Gardner G. J., Sonoda Y., Chi D. S., Barakat R. R. (2009). The incidence of isolated paraaortic nodal metastasis in surgically staged endometrial cancer patients with negative pelvic lymph nodes. *Gynecologic Oncology*.

